# Atmospheric negations: Weaponising breathing, attuning irreducible bodies

**DOI:** 10.1177/02637758231203061

**Published:** 2023-10-30

**Authors:** Mikko Joronen

**Affiliations:** Tampere Institute for Advanced Studies, Space and Political Agency Research Group, Tampere University, Finland

**Keywords:** Atmospheric violence, attunement, breathing, negative geographies, ontology, Palestine, materiality

## Abstract

This paper elaborates various ways in which atmospheric negations operate by weaponising bodily vulnerability to air. It shows, firstly, how bodies remain exposed to colonial proximities of respiratory, olfactory, and sonic violence with ways that are constituted through negating site- and body-spheres. It highlights these spheric materialities by discussing the use of tear gas and skunk water, bombing of chemical warehouses, and the sonic settler aggression in Palestine, further arguing that we need to pay more attention to the irreducibility of the body to such violent orchestrations of atmospheric. Here the irreducibility of the body and the incapacity of the spheric become key matters related to what is called, secondly, the reciprocal sphereological vulnerability between the corporeal and the spheric. By paying particular attention to difference between breathing and attunement, the paper shows how a negative limit condition resides at the heart of what constitutes sphere-dwelling. Here an ontological shift in thinking atmospheric is suggested, one that starts, not from current framings aligned around the notions of vitality, affirmation, and relationality, but from the weaponisation of the fundamental incapacity of the body to overcome its own vulnerability to the air it breathes.

In Spheres trilogy, philosopher Peter Sloterdijk (2011, 2014, 2016) explicated his novel approach to human dwelling and comportment towards the world through the spheres we inhabit. Starting from the ways in which bodies dwell in the spheric intimacy of the womb, Sloterdijk expanded his ‘sphereological’ thinking from the intimate microspheres of subject formation to the macrospheric theory of globalisation – to the planetary ‘foam’ – eventually elaborating the philosophical nature of the volumetric interconnectedness of various ‘vault systems’ as what he referred to as the ‘sphere-dwelling’ (see Elden, 2012). With the notion of ‘atmoterrorism’ in particular, the historical emergence of which Sloterdijk (2009b, 2016) discussed through the variety of events that link to the use of poisonous gases in the First World War, development of pesticides and insecticides, and the use of gas chambers in executing death penalties in US and China and by the Nazi regime, Sloterdijk further underlined the way in which breathing becomes the primary target terrorised with (often lethal) use of force (see Nieuwenhuis, 2016, 2018). Although the idea of targeting, not only the enemy body but the air they breathe, moves the target of violence to spheric environs and proximities of aerial materiality, importantly the spheric also consists of more than a mere replacement of the ‘solid’ (earth) with the ‘gaseousness’ (air). This is evidenced by the discussion of sonic intimacy and terror (Safa, 2022; Sloterdijk, 2009b), albeit it is the notion on ‘atmosphere’ that perhaps at best captures the spheric as something accompanied with vibes, affections and ambiences (see Fregonese, 2017; Hitchen, 2021; Stewart, 2011). Here the scope of sphere-dwelling expands beyond gaseousness materiality that bodies breathe, thus containing various elements that align with, call for and catalyse certain embodied attunements.

 Within existing geographical literature, the affective and material aspects of atmospheres (McCormack, 2008) have been elaborated through various ways in which, for instance, ‘austerity measures’ (Hitchen, 2021), ‘urban security/terror’ (Closs Stephens et al., 2017) and ‘gender violence’ (Khan, 2023) produce various affective atmospheres, or atmospheric materialities shape things from politics of ‘drought’ (Savelli et al., 2022) and ‘dust’ (Zee, 2021) to ‘animal atmospheres’ (Lorimer et al., 2019) and ‘political economies of volumetric violence’ (Mostafanezhad and Dressler, 2021). Herein, however, I want to turn the focus on the *embodied* side of spheric violence, particularly on how the vulnerability of the body to the proximity of air can be used as a locus for looking at material and affective configurations of atmospheric violence through two key modes of embodiment: *breathing* and *attunement*. With examples drawn from the colonial context of Palestine on the use of tear gas and skunk water, military targeting of chemical warehouses, and various sonic forms of terrorising the everyday life, I will explicate, firstly, how the weaponisation of breathing and attunement mobilises bodily vulnerability to spheric. I show in particular how the exposure of the body to negating aerial surroundings operates, not simply through the capacity of the atmospheric to engender violent affective and material forcings of the body (e.g. Anderson, 2014; Closs Stephens, 2016), but rather through the bodily vulnerability to various atmospheric compositions that are, as underlined by feminist geographers for a long, embodied and intimate in nature (e.g. Hyndman, 2019; Jackman and Brickell, 2022; Koopman, 2011; Sharp, 2021). It is such vulnerability, I argue secondly, that ultimately makes dwelling a spheric inhabitation of air: it exposes bodies to atmospheric proximities, but also the spheric to embodiments. On the one hand, vulnerability so engenders reciprocity: it makes breathing bodies vulnerable but also irreducible to atmospheric proximities (and their weaponisations). On the other hand, vulnerability helps in comprehending the negative condition of what ultimately makes breathing bodies sphere-dwellers: namely, the corporeal vulnerability engendered by the impossibility of the body to not breathe (Sloterdijk, 2009a).

I start the paper by focusing on weaponisation of breathing. In the first subsection, my aim is to offer a reading of atmospheric as *site-spheres*, where colonial violence operates by exposing breathing bodies to sites accompanied with disruptive and disengaging respiratory violence of poison clouds that, importantly, also catalyse more abiding atmospheric attunements. Here the focus remains on Israeli military’s use of tear gas and bombing of chemical warehouse, particularly on how certain ‘hotspots’ of aerial violence operate in long term by exposing bodies to repulsive and toxic atmospheres. The second subsection then moves on to look at ways in which the use of ‘skunk water’ – a chemical liquid planned to quell protests through repellent and sticky smell – contaminates spheres of moving bodies, or what I call kinetic *body-spheres*. By discussing the malodours that stick on bodies, I show how bodies become violently disengaged and cut off from their mundane surroundings, thus constituting kinetic olfactory spheres marked with odorous contamination and colonial otherness. The end part of the subsection discusses some of the ways of ‘cultivating the breath’ with rhythms and attunements different of those of atmospheric weaponisations, thus paving the way for a second section, that further explicates the irreducibility of bodies to atmospheric weaponisations. In the second section, the focus is turned on sound, the section further highlighting how the spheric remains vulnerable to bodily *irreducibilities* that push the spheres of sonic violence ‘back to the background’. The final third section then opens up the onto-political ramifications of thinking atmospheric weaponisations (and the negations they carry along) through the vulnerability of sphere-dwelling. Here the difference between breathing and attunement remains a key one: while the attunement to atmospheric negations is shown to disclose a reciprocal vulnerability between bodies and spheres (i.e. the vulnerability of/to spheric), breathing further reveals a *negative condition* of what ultimately makes living bodies dwellers of air-spheres: the limit to corporeality imposed by the impossibility of the body to not breathe. I further elaborate this through the connection atmospheric negations have to what I call the groundless negativity of air. This, I show, forces us to rethink some of the prevalent notions in thinking materiality, affect and politics – relationality, vitality and affirmation in particular – which I will further think with the emerging body of works on ‘negative geographies’ (e.g. Bissell et al., 2021; Dekeyser and Jellis, 2020; Joronen and Rose, 2021; Landau-Donnelly and Pohl, 2023). I conclude by suggesting a novel way for approaching negativity, not as an alternative ontology, but as an alternative to ontology.

## Weaponising atmospheres: Pneumatological proximities

As breathing entities, we dwell by inhabiting the air (Irigaray, 1999). Breathing bodies need air, or oxygen, to live, inasmuch as weaponisations of air need, in order to remain hostile, coercive or lethal towards the body, an entity fundamentally reliant on what remains in its aerial proximity. Importantly, such vulnerability to air is never solely about the biological functions of respiratory organs, such as lungs, but also defines life at more fundamental level. Life begins and ends with a breath, while breathing also participates to a wider process of circulating matter through bodies. In such process, breathing allows the world to become part of what is inside of it (the body), while also letting the body to be so traversed through by what is produced by other entities – namely, the oxygen produced by plant photosynthesis. It is precisely such material immersion, or what Derek McCormack (2008, 2018) calls the ‘envelopment’ of gaseous body, that Emanuele Coccia (2019: 138–139) acknowledges as essentially distinct from the ‘cultural reductionism’ that treats atmosphere(s) as a ‘shared reality of the perceiver and the perceived’ and thus as a ‘cultural fact’ – as a spatialization of socio-cultural ‘climatization’ that, in very Kantian manner, prevents the direct access to the natural world. Coccia blames Sloterdijk’s reading of ‘spheres’ in particular on the ‘gnoseological reduction’ of atmospheres to a metaphysics that effectively hides from a view more elemental climatization process, where the inhaling and exhaling body becomes part of what other entities (plants) produce for the body to remain alive (via breathing). By following this material climatization process, the spheric vulnerability of the body can be seen to contain an aerial element, which through the geohistorical formation of earth’s atmosphere has eventually engendered a planetary material environ that breathing bodies are immersed in and so dependent on as living entities.

Albeit dwelling in atmosphere, bodies nonetheless breathe and sense through more intimate spheres of dwelling. Tear gas, for instance, might disrupt the process of breathing, in as much as a sudden loud noise can disconnect bodies from their immersed practices, yet these disruptions do so by coming near and proximate. It is by taking such embodied aerial proximity as my guide that I want to rethink the two key aspects of atmospheric – breathing (material embodiment) and attunement (affective embodiment). Such, I argue, is a way of thinking the material politics of sphere-dwelling – the politics of inhabiting the air through the spheres of dwelling – through what is ultimately seen as its negative condition: namely, through the inability of the body to not breathe. In as much as breathing hence makes bodies vulnerable to the aerial proximities (the spheric), breathing in spheres is always constituted through (the politics of) what so becomes proximate to bodies.

Importantly, with such proximity I do not intend to refer to vault-like spheric immanence indicated perhaps most clearly by the ‘bubble’ and ‘foam’ metaphors of Sloterdijk (2011, 2014, 2016). Proximity herein is neither metric but resembles the notion of aerial and material intimacy (Sharp, 2021), hence characterizing sphere-dwelling that is constituted, instead of rounding cultural environs, through vibrating ‘shockwaves’ (Fregonese, 2021; Safa, 2022), hovering ‘meteorologies’ (Mostafanezhad and Dressler, 2021), radiating ‘material fluidities’ (Nieuwenhuis, 2016), and olfactory lingering of violence. Such material and embodied understanding of atmospheric proximity thus helps in acknowledging manifold ways of weaponising the air through the necessity of the body to breathe in its aerial surroundings (Sloterdijk, 2009a), but importantly, also in avoiding masculine, techno- and Eurocentric remoteness that many (e.g. Griffiths, 2022; Harker, 2014) have seen problematic to works elaborating aerial violence through its volumetric and vertical dimensions (cf. Adey, 2010; Weizman, 2007). Such is not merely of turning the focus from a ‘view from above’ to a ‘view from below’ (Adey et al., 2011; Billé, 2023); it is rather a way of comprehending the spheric, and the volumetric dimensionality it indicates, through the *pneumatological* proximity of air. While in a case of colonial violence in Palestine this might mean, as Christopher Harker (2014) writes, that attention needs to be paid to the life of those to whom the weaponisation of air is all but remote, it further resonates with the bodily intimacy many feminist geographers have addressed as a key for comprehending politics (e.g. Hyndman, 2019; Sharp, 2021; see also Legg, 2023). Indeed, breathing is not only passive inhaling that receives poisonous gases lingering in its spheric nearness; it is also about exhaling and interaction that produce material and affective spheres, as the COVID-19 pandemic has exemplified, through the pathogenic contamination of air and the fear of breathing the same air with the others. While the views of Sloterdijk (2009a, 2009b) and others (Adey, 2010; Nieuwenhuis, 2018) on ‘atmoterror’, ‘airquakes’, ‘gaswar’ and so on hence do help in underlining how environs are transformed into negating respiratory spheres, the body is never reducible to a mere carnal disposal site of lethal gases. Bodies escape and nullify weaponised spheres, thus creating what I call bodily irreducibility to spheric. It is in this regard that the spheres of air remain incapable of fully capturing bodies – their biological functions, spheric attunements and political sensibilities – within their weaponised configurations of violence.

In what consists of the remaining part of this section, I will turn the focus first on the weaponisation of atmospheric negativity. I will do so in a colonial context of Palestine by elaborating ways in which atmospheric violence operates through the weaponised spheres that disrupt, mark, and attune bodies in their mundane spheres of dwelling. Focus on everyday atmospheric intimacies in such extreme context of colonial violence, I claim, underlines the irreducibility of life to power, and thus the ways in which Palestinian bodies, despite the brutalities of violence, nonetheless ‘cultivate breath’ with rhythms and attunements different to those of weaponised atmospheres. Instead of thus starting from the capacities of various weaponisations with ways that often end up (intentionally or unintentionally) glorifying colonial abilities to dominate – at best framing subaltern populations through epiphenomenal ‘resistance’ (Harker, 2011), ‘suffering’ (Hammami, 2015), ‘victimhood’ (Joronen, 2017) and ‘traumatisation’ (Marshall, 2014) – through spheres of intimacy the focus can be turned, already at start, on *pneumatological proximities* of atmospheric weaponisations. Importantly, such approach helps unearthing various, often overlapping forms of violence through embodiments irreducible to atmospheric configurations they contain.^1^

### Site-spheres of poison clouds: Dwelling at hotspots

In 2022, Israeli police used drones to drop tear gas to a Temple Mount at occupied East Jerusalem, causing panic among Palestinians finishing the Ramadan Friday pray at the compound. The manoeuvre led to events that wounded 57 Palestinians, including children and elderly, of whom 26 suffered from gas inhalation, 12 of them being hospitalised (Breiner et al., 2022). Indeed, when inhaled, tear gas can cause various incapacitating respiratory symptoms, ranging from trouble breathing, coughing, salivating, and choking to vomiting and, at worst, to respiratory failure. These are often accompanied with various eye and skin symptoms, from tearing and temporary blindness to itching and chemical burns (Brown et al., 2021). Depending on spheric matters, such as the time and closeness of exposure, or openness of the exposed area, and various physiological features, such as pre-existing medical conditions, such atmospheric violence can lead to severe pain-impulses, involuntary processes on the glands, autonomous reflexes on muscles, and altered breathing patters, that together incapacitate the body to varying degrees (Bessac and Jordt, 2010).

As the events in Temple Mount exemplify, tear gas, originally developed as a weapon of war and (colonial) policing (Feigenbaum, 2017; Legg, 2007), operates by engendering gaseous disruptions that use the respiratory necessity of the body to breathe to harm, incapacitate and negate it – to govern the body through the weaponised spheres of air. Tear gases, however, do not merely disrupt bodies present in spaces they are used, but importantly also affect those dwelling nearby their spheres of influence. This was captured well in an interview I conducted in 2022 at West Bank, in a site located at Bethlehem governorate. A woman, Nadine, whose husband’s family lives at close vicinity of a site where clashes between Palestinian protesters and Israeli Defence Forces (IDF) soldiers regularly break out, explained the tensions of having a house frequently surrounded by clouds of poison gases. ‘My husband wants us to move back to his family house’, she started, while adding, ‘but it’s a “hotspot”, almost every week there are clashes outside the house’. She went on explaining how her husband grew up in the house and got used to all the violence, even as kid (during the Second Intifada) seeing ‘dead people in front of his home door when leaving to school’. While underlining how for her husband the violence might have become normalised, for her the threatening atmosphere, accompanied with constantly looming possibility of violence, simply felt ‘breathless’. She unpacked:

One weekend we were there [her husband’s childhood home], having lunch, with our kids with us, when we heard tear gas and stun grenades being shot. My mother-in-law just said, ‘they will quit soon’, but I could see she was stressed below the calm. Stressed about soldiers entering the house, about all windows being properly closed, so that the tear gas or the smell of skunk water would not get into the house. If it does, the smell gets stuck, and doesn’t get off just by opening the doors and windows.

Nadine further underlined the consequences of living in such spheric ‘hotspot’ of colonial violence by telling how she can see her mother-in-law being constantly over-worrying all possible things: ‘even when out of the house, she keeps worrying if windows were closed properly’.

Here we are not only witnessing a slow atmospheric violence that ‘wears down and out’ people (Harker, 2020: 161) with enduring ‘vulnerabilities’ (Joronen 2021), ‘environmental damage’ (Amira, 2021) ‘material toxicities’ (Griffiths, 2022), and forms of ‘dispossession’ (Sa’di-Ibraheem, 2020); here we are also getting a sense of how the colonial violence operates through spheres that are both aerial and intimate in nature. Nadine further highlighted this through different positionalities between herself and her mother-in-law:

the place where I grew up is not far away, but soldiers have only once invaded my family house, during the second Intifada. My husbands’ family house, however, has been invaded and searched dozens of times by [IDF] soldiers looking for kids who threw stones and escaped to the garden by jumping over the yard fence.

She recalled another event that took place when she had just met her husband-to-come: they were about to leave her husband’s family house, but after stepping out to the street realised they were caught between gathering protesters and armed soldiers. ‘The situation was about to explode, and when we tried to quickly walk away, I got hit by a stone … soldiers started shooting tear gas and more stones hit the air. It’s almost like living in a different world’, she concluded, again comparing the site to her own childhood home less than a mile away.

As the events above exemplify, the use of gaseous ‘demonstration control agents’ brings forth disruptive negations that, by turning homes, streets, and sites of worship to spheres of spectacular colonial violence, profoundly affect everyday life. And yet, it is also the case that here the spheric gaseousness turns from an abrupt disruptive event to a more abiding atmospheric *attunement* accompanied with constant stress, alertness, fear and worry that together constitute peculiar ‘sphere-worlds’ that differ from sites only few blocks apart from one another. Atmospheric, in other words, engenders intimate and proximate site-spheres of aerial exposure, but also stretches over time when constituting long-term spheric attunements to aerial configurations of ‘slow’ colonial violence. ‘My husband’s family has had the house for several generations’, Nadine added a bit later, further explaining how before there were ‘no walls and no protests’, and how today much of the agricultural lands the family used to grow olive trees and spend leisure time are now stolen and annexed to settlements. ‘I understand they [her husband’s family] want to continue living in the house’, she concluded, while adding words that further underline the affective and atmospheric proximity of everyday colonial violence: ‘but I feel unease there: I just cannot bring my kids to such a place with constant tension coming from the surroundings”.

In a similar vein, another interviewee, Halima, talked about the challenges of raising her small daughter in a nearby West Bank village Israeli soldiers constantly enter, often by throwing tear gas and stun grenades without any prior warning or visible reason. She explained:

once the tear gas came into our house. My little daughter was scared and started crying – it was really bad for her eyes! This puts an extra pressure on us as parents to try to give our children a safe childhood.

She then concluded: ‘its fear, continuous fear of raising a child here. The violence is everyday, constant, spontaneous – I didn’t have this in my childhood’. On another occasion, Samir, close to his 40s, talked about his old aunt, who lives alone near one of the ‘hot spots’. Samir showed his aunt’s house that, as he put it, had ‘become like a Guantanamo prison’: barbed wire everywhere, windows always closed and covered with metal protectors (some with bullet holes), and tall brick walls with sharp spike bars at the top surrounding the house to prevent both, protesters seeking shelter and the soldiers chasing them, from entering the premises.

 Atmospheric violence, it seems, is not only mediated through spheric elements related to abrupt events of disruption; through repetition it also engenders more enduring atmospheric negations, which attunements and materialities become markers of everyday life in certain site-spheres of dwelling. This can be further exemplified with events that took place in the urban area of Beit Lahiya during the 2021 Gaza war. In 15 May, IDF missile strike set in fire the largest agricultural chemical warehouse in Gaza (Khundair Pharmaceutical and Agricultural Tools) filled with hundreds of tonnes of pesticides, fertilisers, and other farming materials. The fire engendered a toxic cloud over the area of around 6 square kilometres, where the air concentration of chemicals crossed the ‘acute emergence levels’, posing a high risk of irreversible damage to human health (Al-Haq, 2022). Since the events, which according to the report conducted by Forensic Architecture at Goldsmith (University of London) and human rights NGO Al-Haq are ‘tantamount to the use of chemical weapons through indirect means’, residents of the contaminated area have been reported to struggle with several health issues, but also, as more people have gotten sick, to increasingly worry for their health (McKernan and Balousha, 2022).

As the examples above show, atmospheric negations remain entangled to certain *site-spheres* of colonial violence, where the atmospheric negations become entwined to both, to breathing (in contaminated site-spheres) and attunement (to violent spheres of gaseous toxicity). And yet, as I will discuss in the next subsection, such weaponisations also operate by contaminating bodies through spheric violence that is more kinetic in nature. Such moving *body-spheres*, and the olfactory functions that move along the bodies, can be further exemplified with a use of Israeli-produced demonstration control tool: the skunk water.

### Kinetic body-spheres: Breathing skunk, cultivating breath

‘Skunk water’, an industrially manufactured malodorant liquid originally developed for quelling protests, contains a rotten and repellent smell that gets stuck to bodies, clothes, and spaces it lands to – streets, homes, shops, balconies, hair, tarmac, etc. Fired from a water cannon placed at the top of armoured vehicles (or alternatively dropped from bags carried by drones), it has been widely used by the IDF and Israeli Police, often to spray vast areas, even entire Palestinian villages and neighbourhoods, under a thin pretext of security (e.g. ACRI, 2014). When fired, the liquid transforms to a mist that converts the air to a putrid totality that affects everyone inhaling or otherwise in touch with its aromatic malignance, causing immediate reactions of gagging, nausea, and vomiting. Israeli military, law enforcement, security companies and the manufacturing company, Odortec, praise skunk water as non-toxic, ecological, humane, ethical, and organic alternative (according to the manufacturer, ingredients consist of baking powder and yeasts synthesized with amino acids) to other crowd control weapons, such as rubber bullets and tear gas (see B’Tselem, 2013; Odortec, 2012). However, those inhaling or being hit by the extremely foul-smelling liquid describe it to resemble a mixture of ‘rotting animal corpse’, ‘open sewage’, and ‘excrement’, the aroma of which can stick to bodies, objects, and environs for weeks, even for months (see Levy and Levac, 2021).

Skunk water, however, does not merely quell protests through a weaponization of air; by getting stuck to what it lands on, it also contaminates spheres of living, thus offering a tool for collective punishment. Especially, when sprayed in urban environments, the skunk water can affect lives of thousands for days through the repellent cloud-sphere it creates. When repeatedly used in certain hotspots, often together with tear gas and stun grenades, it also engenders negative atmospheres resembling to those discussed above as site-spheres of tear gas ‘hotspot’. Skunk water, however, also marks the sphere of bodies it hits through the smell that gets stuck to them. It is in this regard that skunk water signifies more than an abrupt control tool used for incapacitating violent mobs: it cuts off sprayed bodies from their mundane environments through its fetid aromatic marking, while further torturing them with a repellent smell that sticks to bodies. As a sticky putrid smell, it does not only mark certain neighbourhoods or make environments it lands on repulsive to dwell, but also separates bodies it hits from their spheric surroundings.

It is here that we can witness another way weaponised spheres constitute embodied environs; namely, through the foul odor exuding and spreading from moving bodies contaminated with the material scent of skunk water. By contaminating the body-spheres, such odorousness marks colonised bodies, while amputating and cutting them off from their everyday spheres of co-dwelling. As one interviewee put it sarcastically, when asked about the effects of skunk water, ‘if you get sprayed, nobody wants to meet you’. Here the body itself becomes sphere-contaminating to the extent that its olfactory amputations should be seen in a broader context of racial manufacturing and dehumanization of colonised bodies. The mundane effects of such marking are well exemplified by Franz Fanon (1967) in his postcolonial classic, *Black Skin, White Masks*, where Fanon describes the negating ‘nausea’ of being a racialised black body in the ‘white world’ – of being constantly pointed out, separated, disrupted, prevented, disturbed, and negatively bonded to mundane spaces of dwelling. Similarly, foul-smelling colonial bodies remain obnoxious for weeks, being thus negated from their spheric surroundings, not through the racialised visibility of the skin, but through the humiliating and dehumanising othering. Such aromatic amputation, in other words, offers a peculiar atmospheric technique of incapacitation: it separates bodies from their spheres of co-dwelling by turning them unhomely in their mundane spheres of dwelling. The spheric, now recentred from site-spheres to the kinetic (mal)odorousness of the body, becomes a realm of aromatic negation, where the foul body exudes its own exclusion, humiliation and marked colonial otherness.

Admittedly, the olfactory marking transforms bodies from mere corporeal inhalers of foul-smelling and harmful gases to mediators of aerial contamination and fetid humiliation. Here material and affective atmospheres, and the breathing and attunement, become complexly tied to one another. This was further exemplified through series of workshops I organised in altogether nine rural West Bank communities together with a local NGO offering free low-level preventive psycho-social education. One of the participants described past events that still made it difficult for her to *breathe*. Short of breath already when sharing the emotionally burden events, the woman, in her mid-30s, explained the humiliation caused by the violent night raid of IDF. She described the way Israeli soldiers had entered the village and unexpectedly broke into her home in the middle of the night, dragging her (along with her family) out to the street under dressed. The woman, Nadia, told how being at street in little clothes and without her hijab on, while the soldiers raided the house, was extremely humiliating in front of a small village community. She further added that she nowadays often ‘goes to bed with her hijab on’ if there is a ‘tension in the air’. Importantly, she was seeking guidance for the way such stigmatising humiliation, constant worry and anxiety had given her a trouble breathing in her most intimate everyday sphere of dwelling.

The way the military raid had turned the home and the everyday life of Nadia into a negating sphere makes evident how breathing is closely related to certain atmospheric attunements. Like in sites of constant disruptive use of tear gas and skunk water, here as well negating atmospheric attunements turn those dwelling in them, literally, breathless. Atmospheric becomes more-than-air-mediated, breathing in turn connecting bodies to certain negating atmospheric attunements. And yet, breathing is not only receptive – a way of breathing in certain atmospheric attunements – but a way of *breathing back* to negative atmospheric surroundings. This was tangibly present in ways that the organisation I worked with organised breathing exercises that people in our co-organised workshop groups also tend to find most helpful in dealing with everyday negating atmospheric attunements – stress, fear, humiliation, constant alertness, etc. ‘The breathing and relaxation are practical techniques to deal with anxiety’, my collaborator started while going through various socio-political situations and the consequences of long-term exposure. Here ‘being breathless’ is no more a mere figure of speech or a metaphor for emotional load: people living in villages under constant military and settler violence ‘suffer from pressure on their chest, they feel their chest is heavy, and that they can’t breathe properly’. After describing the load of continuous exposure to violence, she then gave an example on one of the situations where breathing techniques have been particularly important:

We also taught this breathing technique to children and teenagers who are detained. When you are detained, and you are in handcuffs and you cannot move, it’s good to breathe, because it helps you for decision making – to not give reactions, to help you to just calm down, or simple not to faint and panic.

Importantly, such ways of ‘breathing back’ can be seen to contain, as Marijn Nieuwenhuis (2015) has suggested by following Luce Irigaray’s (2002) idea on ‘cultivation of breath’, politics and knowledge on how to resist, or better, not to reinforce the atmospheric compositions of colonial violence. Here the air becomes elemental to embodied politics, the *cultivation of breath* offering not only a personal autonomy over the pace of breath imposed by violent atmospheric configurations, but also a possibility for a different attunement. As the example above shows, bodies breathe different attunements: they learn to breathe in rhythms different to those of occupation, so making breathing a transformative moment – a moment of transforming the atmospheres of colonial violence. Such ‘cultivation of breath’ is not merely a way of protecting the biological functions of the body from the toxic and contaminated air, for instance, with a mask (as done by some Palestinians protesters in cases of tear gas use and skunk water spraying). Breathing rather becomes a transformative moment, through which bodies, by undoing the atmospheric weaponisations, breath beyond the prevalent modes of attunement and atmospheric negations. It is to this non-affirmative aspect that I will turn next into.

## Vulnerable spheres: Irreducibility to sonic

Considering the above, bodies should be seen irreducible to what they at the same time fundamentally depend on; namely, the proximity of air. In this section, I want to further exemplify such *irreducibility* by turning the focus shortly on sonic violence and its relation to attunement. Such is not to force out a marginal aspect of atmospheric violence in Palestine: after Israel’s withdrawal of settlements from Gaza Strip in 2005 and the start of the blockage policy in 2007, for instance, the corporeal presence of military and settlers in Gaza has been replaced by a constant buzz of drones patrolling the besieged airspace. Indeed, as a ‘background hum’ drone noise repeatedly penetrates everyday sonic spheres and the aerial environs of Gaza, and hence shows how sonic spheres are not only important for studying multisensory embodiments in/with the world (e.g. Gallagher et al., 2017; Simpson, 2016), but also relate to complex relations of power and violence (e.g. Goodman, 2010; Paiva, 2018; Safa, 2022). As embodied, sounds constitute intimate spheres of hearing and vibration, which in modern times have become filled with machinic terror of signals, alarms, and various forms of (technological) noise constantly humming at and coming forth from the background. Gazan airspace alike is not only occupied by the surveillance drone buzz, but also by frequent vibrations of low flypast fighter jets that engender shocks with ‘reverberations’ (Safa, 2022) accompanied with long-lasting affective repercussions. Importantly, these modes of sonic disruption are intimate and shared in nature: they might attune bodies differently to varying thanatopolitical threats, at the same time engendering shared embodied communards of noise-terror.

Sound can hence create threatening atmospheres, where vibrations and noise terror are embodied and shared through varying bodily attunements. This was well exemplified by a situation which took place during my fieldwork in one of the rural West Bank villages. The situation started to escalate during the olive harvest, which, as always, was a family event, as I was also reminded by a man who had come to help his parents with the harvest from a nearby town. After the day’s work, we started walking downwards the olive grove to visit a spring nearby. On our way down, a quiet music reached us, first staying at background, as if coming from a distance, only occasionally becoming louder and catching my attention. I recalled thinking the music must have come from a car of local youth moving the streets of the village located uphill the valley we were at. As we finally reached the springs, the vibrations of pumping techno-beat suddenly became very loud and were accompanied with overtly aggressive choir of screams. One of the villagers must have seen my puzzled look, as he mentioned ‘it’s settler music’, adding that the settlers were gathering further down the valley. For a moment, the tension was palpable, as if everyone were taking a deep *breath*. And yet, the impact on local families was almost null: children continued to laugh and play around the water pool next to the spring, no precautious acts were made or worrying looks changed, no signs of deep concern were visible – only the atmosphere had suddenly changed, the adults being, at least for a short moment, a degree quieter. For a scene that was filled with sonic aggression marking the presence of settlers in a site only recently excluded from the daily lives of West Bank villagers, everything seemed to continue as if nothing spectacular had happened.

As the events discussed above exemplify, air-terror of aggressive noise might have been spheric and capable of transforming the atmosphere at the moment, yet without being able to significantly alter enactments. Such atmospheric transformation was not simply passing through bodies without noticing; it was rather embodied by taking a long breathe that drained empty its sonic terror. When I returned to discuss the events later on, one villager stated that ‘we need to stay strong’ and ‘not to let these things to get under our skin’. It is precisely such indifference, the ‘need to stay strong’, that hides a key ambiguity. On the one hand, the momentary attunement to indifference constitutes something rechannelling the atmospheric violence of sonic terror. Though incorporating the everyday and changing the atmosphere on the spot, the noise was nevertheless pushed *back to the background*. On the other hand, it is precisely through these indifferences that the atmospheric also did channel attunement. The manifoldness of becoming attuned, in other words, brings to the fore an ambiguity at the heart of sphere-dwelling: it testifies how spheric violence can be engendered through negating sound-spheres that channel attunements and re/inactions, while simultaneously underlining the inability of the spheric to dictate what it so catalyses. Spheric weaponisations, in short, constitute bodies without being able to reduce them to their coercive functions and terrorising attunements.

It is precisely this ‘inability to reduce’ that I argue signifies, not only the ‘ungovernability of the body’ (Joronen and Griffiths, 2022), but crucially also the incapacity of the spheric. To a certain extent, bodies are always vulnerable to atmospheric alterations: they are constituted in and through the spheric and its atmospheric (material/affective) compositions. And yet, vulnerability never signifies a mere vulnerability to spheric, but also a vulnerability of the spheric: namely, the incapacity of the atmospheric weaponisations to orchestrate life to what it conjures as its ambient surroundings. It is precisely for this reason that the focus in studying inhabitation of air should remain, not only on those ways through which the spheric constitutes bodies, but on those irreducible vulnerabilities through which bodies become attuned to atmospheres (see also Bille and Simonsen, 2021). The body, and its irreducibility to negating spheres, becomes a key entry point through which to understand sphere-dwelling as inexhaustible to, and yet constituted through spheric configurations of violence. Such is not simply a sign of the vulnerability of the body to the spheres it remains exposed to; it also offers a window for thinking the fragility and incapacity of the spheric. Sphere-dwelling, in this regard, denotes *reciprocal vulnerability* of bodies and spheres.

## Groundless air: On negative condition of sphere-dwelling

As the discussion so far has shown, sphere-dwelling names a reciprocal vulnerability between the body and the spheric. It is this negative condition of inhabiting the air – the vulnerability of/to spheric – that atmospheric weaponisations mobilise without being able to fully exhaust bodies to their violent orderings. In this last section, I want to think further on the onto-political ramifications of such prevalence of the negative in sphere-dwelling. I will do so by asking what the atmospheric negations discussed above, with their disruptive, contaminating, marking, amputating, disorienting, repulsive, humiliating, and incapacitating functions, can unfold about the material and affective aspects of inhabiting the air. My aim is to open up a space for thinking the ramifications of atmospheric negations, particularly the questions of vulnerability and irreducibility they were shown to instigate, through what I call the breathing of *groundless air*. I will do so by problematising three ontological aspects that over the last 20 years or so have become paradigmatic for approaching materiality, affect and politics in geographical literature (and beyond): namely, relationality, vitality and affirmation. While acknowledging that these aspects do not always travel together (see Mol, 2013; cf. Coole and Frost, 2010; Ojakangas, 2005) and that they do carry versatile, even opposing takes within (see Bridge, 2020; Pohl, 2021; Roberts, 2014; Roberts and Dewsbury, 2021: Saldanha, 2020; Shaw and Meehan, 2013), they nevertheless emerge, I show, less unproblematic in the light of the negative condition of sphere-dwelling.

To unpack this, we need to go back to a difference between breathing and attunement. While the latter refers to ways of becoming-attuned to certain atmospheric configurations, breathing is importantly also about staying alive in certain gaseousness formations of material atmospheres. Albeit both do help in acknowledging our bodily vulnerability to atmospheric, it is the latter that brings to the fore the irresolvable limit present in all corporeality: the impossibility of a living body to not breathe. As Luce Irigaray (1999: 8) asks, ‘is not air the whole of our habitation as mortals? Can we live elsewhere than in air?’ Indeed, body dwells in spheres by breathing. While inhaling and exhaling are something that take place in gaseous proximities and in relation to various (at times toxic and weaponised) compositions of air, the necessity to breathe is in itself something the body remains dependent on, and, as a result, fundamentally limited by. Breathing is something that demands to be constantly addressed, thus constituting, as Joronen and Rose (2021: 1410) recently wrote, a vulnerability to ‘which our bodies are wholly and unremittingly beholden’. Corporeal vulnerability to breathing, in other words, signifies a condition that a living body *cannot* resolve, escape, or overcome. Breathing in this regard constitutes a negative condition that constantly makes bodies dependent on air, and so exposed to the aerial proximities they inhabit.

Importantly, it is this unresolvable vulnerability to air that, I argue, reveals the intrinsic negativity of sphere-dwelling. Such dependence between the body and the air is not something that could be framed merely as a *relational* assemblage, ultimately constituted out of the relational field of (always) capable constellations of affective forces. Sphere-dwelling rather denotes a sphereological vulnerability that is fundamentally limited by the bodily inability to overcome its own vulnerability to breathing. While ways of weaponising breathing do certainly contain differences that go back to various (power) relations (as exemplified in previous sections) that also constitute breathing bodies in relation to entities other than human – gases, molecules, sound waves, weapon technologies, climatic alterations and so on – there is no relational configuration of ‘sphere-dwelling’ that could somehow set aside the corporeal necessity to breathe. As Paul Harrison (2007: 591) writes, with a nod to Heidegger (2001), inasmuch as ‘the hollow [or should we say the *air*?] gives the essence of a jug, the nonrelational relates the relational’ [my addition]. All relational assemblings and weaponisations of sphere-dwelling are, in other words, carried by the *nonrelational* facticity that in order to be alive, the body needs air to breathe.

It is here that we arrive to what remains a key for understanding how atmospheric negations work as embodied proximities of sphere-dwelling. Atmospheric negations orchestrated with tear gas, skunk water, and poison clouds, or through hostile attunements, are ways of weaponising bodies through their vulnerability to spheric. Importantly, this comes back to the very possibility of bodies to orientate towards the world, as atmospheric weaponisations, instead of successfully directing bodies towards the spaces they dwell in, constitute spheres that disrupt, contaminate, incapacitate, wear out, and cut off bodies from their (mundane) orientations (Hannah, 2019). Here the focus turns from what bodies are capable of to what they are incapable of, which further problematises the inherent vitalism and ontologisation of capacity that characterises much of the existing discussion on affect and materiality (e.g. Anderson, 2014; Bridge, 2020; Gallagher, 2016; Roberts, 2014; Roberts and Dewsbury, 2021). Atmospheric weaponisations create what Sara Ahmed (2006) aptly calls the ‘spaces of I cannot’ – spheres where bodies remain first disoriented, disrupted, prevented, incapacitated, violently negated, or even completely amputated from their ability to orientate towards the world.

In as much as bodies hence remain incapacitated by atmospheric negations, it is also the case that the spheric too remains vulnerable to bodily functions, enactments, and sensibilities. Bodies can learn to ‘breath back’ and ‘cultivate breath’ with rhythms different from those of violent atmospheres, in as much as they can find ways of attunement different to those dictated by atmospheres of aggression. Importantly, the affective politics of such bodily irreducibility cannot be reduced to a mere valuation of joyful and hopeful affectual registers over the negative and paranoic ones (Sedqwick, 2003; cf. Hitchen, 2021; Ruez and Cockyane, 2021). Sphere-dwelling rather consists of irreducible embodiments, where bodies might remain exposed to certain atmospheric attunements, but where they simply learn not to affirm. Atmospheric weaponisations, especially when potentially lethal or otherwise posing a serious threat to life and health, thus bring forth the political importance of *not affirming*. As shown above, such politics is also connected to breathing – to a cultivation of breath with different rhythms; to a protection of breathing; even to a moment of ‘taking a breath’ when the disruptive sonic aggressions fill the ether. Here bodies are not simply negating the atmospheric negations in symmetric fashion, but instead *cultivate their irreducibility* to spheric violence by breathing beyond (and against) the prevalent atmospheric weaponisations. Bodily irreducibility to atmospheric hence works, not in a dialectical way (see Mann, 2008; Sheppard, 2008), but by mobilising the irreducibility of the body to what the body inhabits.

It is this irreducible sphereological reciprocity that resituates ‘sphere-dwelling’ from the ontological orderings of affirmative vitalism to what I call the negativity of the ontological. Sphere-dwelling shows how atmospheric negations are constituted through a sphereological vulnerability that further marks the incapacity of the spheric and the irreducible vulnerability of the body. Importantly, such acknowledgment offers a reading of negativity as a sphereological vulnerability that does not define, order, or ground ontological configurations of atmospheric but merely pushes a negative limit-figure at their centre. Instead of ‘double yes’ of affirmative thinking, where according to Paul Harrison (2015) the ‘first yes’ recognises the ontological primacy of productive becoming of life, the ‘second yes’ presenting a priori ethical judgement that prioritises the ‘affirmation of affirmation’ as the good, and indeed, the only preferable ethical choice for politics, such limit-figure allows various constellations of sphere-dwelling to come to being as compositions incapable of draining empty their own negativity – their own vulnerability. Here the ‘first yes’ to ontological liveliness and productivity becomes problematic through the prevalence of negative condition of ‘sphere-dwelling’, which constantly keeps breathing bodies exposed to spheric, and the spheric unable to maintain its atmospheric configurations. The ‘second yes’, the preferred ethical judgement, in turn is left open to various cultivations of irreducible bodies that, often in order to merely stay alive, need to undo, escape, and even end (Dekeyser, 2023), rather than affirm, atmospheric weaponisations.

It is regarding the first yes (to ontology) that I suggest the recognition of breathing as a pneumatological vulnerability can help in turning the focus from the ground-offering onto-theologies and ontologisations of positive metaphysics towards thinking sphere-dwelling through its dependence on what Luce Irigaray (1999: 5) calls the ‘groundlessness’ of air. By following Irigaray’s insight, instead of simply serving a ground for living, air can be seen to point out the impossibility of final ontology; namely, its negativity. It does so, not necessarily through the jug-defining ‘hollow’ of Heidegger (2001: 167) that ultimately signifies, as Harrison (2007) suggested above, the ‘emptiness’ and ‘void’ as part of the ontology (see also Kingsbury and Secor, 2021), but by dissolving the ontological. Air, in other words, is not a thing but a vulnerable happening that constantly allows fluctuating, radiating, hovering and vibrating materialities to emerge and dissolve through its groundless openness. Indeed, as Ingold (2005) reminds, we need air not only to breathe and do things, but also to perceive: air transmits fields of radiant energy and vibrations that allow us to see and hear; it carries (mal)odorous molecules that bodies (with olfactory receptors) can smell; and it holds separations and proximities that allow us to touch and relate things. In other words, the groundless openness of air signifies air’s ability to enable (and disable) seeing in light and dark, hearing in silence and noise, and breathing in gaseous compositions that air brings proximate. What remains proximate in inhabitation of air is thus not something solid with a ground, but the groundless *no-*thingness of air, always ruining meta-physical endeavours in their search for foundations, ground, and abiding ontological axioms for existing (by literally making them to vanish into a thin air).

And yet such air, though constituted through the impossibility to fix the ontological with positive denominators, is also breathable air. As a lack of ground, it opens up air-spheres of proximity for bodies to dwell, breathe and live in. With every breath, such pneumatological proximity of air and its material compositions is being build and undone, this being the case also when such proximity is turned unbreathable with toxic, polluted, and weaponized air-spheres. On the one hand, bodily vulnerability to air can hence be weaponized to transmit formations of atmospheric negations; on the other hand, these weaponisations of air are fragile, constantly at the verge of losing their compositions. Tear gases disperse and vanish in the air, skunk smell wears off and eventually faints its odor, sounds echo and vibrate for their own time in their limited proximities – even long-term affections to violence become replaced with other atmospheric attunements. Air, in other words, can transmit but not ground; it is neither empty (void) nor a thing, but open for the fluctuating, vibrating, radiating, and lingering proximities to happen for bodies to sense, breathe and dwell in.

Importantly, it is such proximate climatization process that speaks further to recent works on ‘negative geographies’ (see Bissell et al., 2021; Dekeyser et al., 2022; Landau-Donnelly and Pohl, 2023), in particular by considering negativity, less as an alternative ontology (i.e. as a negative ontology), and more as an alternative to ontology (i.e. as a negativity of ontology). This is not mere semantics, but a question of approach: transmitted proximities of air do contain world-forming compositions constituted through their prevalent ways of coming to being, but they do so out their proximate happening,^2^ not through the axioms set to define their existence. Groundless air thus does not replace one set of ontological orderings with another, presumable less obsolete one – it does not ground a new ‘political ontology’ (Landau-Donnelly and Pohl, 2023) of air with yet another metaphysical ‘path-dependencies’ (Joronen and Häkli, 2017) at worst promoting Western, masculine and/or race-blind sensibilities (see Ruez and Cockayne, 2021; Sundberg, 2014; Tolia-Kelly, 2006) – but rather allows spheric proximities to emerge, happen and melt into thin air. Indeed, such groundless negativity is precisely what keeps the air open to various atmospheric climatization and weaponisations, while also carrying them in their vanishing ontological compositions and dissolving proximities. Negativity, as an impossibility to disclose sphere-dwelling in ontological terms, signifies what remains transmitted to the proximity of breathing bodies: namely, the groundless air, the compositions of which (lingering and dissolving) the body inhabits, depends on, and remains irreducibly exposed to.

## Conclusions

In this paper, I have offered a reading of the negativity of sphere-dwelling through atmospheric weaponisations of body. By discussing the role of breathing in the constitution of sphere-dwelling, I showed how bodies remain elementally reliant on the materiality of the air, thus engendering what was called the negative condition of sphere-dwelling. A body cannot not breathe, as Sloterdijk (2009a) also acknowledged, this further underlining how the pneumatological proximity of breathing is, on the one hand, nonrelation condition that the body cannot overcome, resolve or undo (Harrison, 2015; Joronen and Rose, 2021), while on the other hand constituting atmospheric proximities, that the body remains vulnerable (rather than affirmative) to and incapacitated (rather than simply capacitated) by. It is such spheric vulnerability to air that various atmospheric weaponisations mobilise as their asset when constituting proximities of colonial, racial, material, and affective violence. These modes of weaponisation were shown to contain various configurations, ranging from prolonged atmospheric attunements of dwelling at ‘site-spheres’ of aerial violence to odorous contamination of ‘body-spheres’ and reverberations of sonic aggression. And yet, they also revealed cultivations of indifferent breaths, different rhythms of breathing, and moments of deep breath, all accompanied with ways of attunement irreducible to those of violent weaponisations of atmospheric. Indeed, for those constantly targeted with atmospheric negations, it is often a necessity to learn to dwell with ways that undo, escape or remain indifferent to their violent forcings.

And yet, while aerial proximities can transmit weaponisations that use the pneumatological vulnerability of bodies to air as their asset, these weaponisations cannot form a ground to what eventually makes them to dissolve; namely, to the groundless air. Recognition of such ontological negativity – the lack of ground in air other than the negating necessity to breathe whatever remains at the spheric proximity – forces us to rethink ways in which notions of vibrancy, becoming, distributed (relational) agency and affirmative politics are circulated in existing literature often accompanied with post- and more-than-human, and (not so) new materialist readings (see Anderson, 2014; Thrift, 2009; Zee, 2021). Herein I have asked how these prevalent ontologisations – especially around notions of vitality, affirmation and relationality – look at from the perspective of atmospheric negations that base their violent and disruptive functions on embodied and negating pneumatological proximities of air. Important in this regard is not only that bodies, as air-dwellers, remain vulnerable to atmospheric proximities, but also that the spheric weaponisations themselves remain incapable of dictating bodies exposed to them. While the latter incapacity (to orchestrate bodies through atmospheric configurations) was shown to remain entwined to bodily irreducibility and the vanishing compositions of groundless air, such negative understanding of sphere-dwelling can also open up new avenues for thinking corporeality and material environments in relation to disruptions, amputations and disorientations associated with colonial, capitalist, racial, imperial, ecological and technological weaponisation of aerial toxicities, atmospheric threats, and hostile attunements (e.g. Griffiths, 2022; Nieuwenhuis, 2016; Sharpe, 2016). In doing so, they can further bridge recent debates in cultural geography (e.g. Bissell and Gorman-Murray, 2019; Dekeyser et al., 2022) with more intimate readings on political geographies of aerial violence, atmospheric subjugation, and colonial/racial weathering (e.g. Collins, 2020). At latter front in particular, focus on pneumatological proximity of breathing, and the violent climatizations its weaponisations constitute, can help in comprehending volumetric violence in more intimate terms and the intimacy more through the material politics of inhabiting the air.

## Notes

1 While more emphasis is given for recent escalations of violence (2021–2022), the paper is based on more than 10 years of fieldwork over which materials have been collected (in/on West Bank, East Jerusalem, Gaza), by using various methods ranging from theme and walking interviews to participatory and ethnographic research, workshops, and archival research.

2 This evidently resonates with Heidegger’s (1972, 2012, 2013) notion of being as an event (*das Ereignis*) that reveals and conceals ontological gatherings for their finite time (Joronen, 2016). Things might always remain ontological in nature, but they are not so out of the status given by intellectual scaffoldings, but rather out of their ontologizing events of ‘being appropriating’ and ‘giving’ its spatialisations and temporalisations. Instead of offering a ground, or a framework, ontology happens, ontologises its compositions. Such a process obviously creates a different task of thought than the one of framing.
